# Supplementary material for: Outcomes of Acute Kidney Injury Among Hospitalized Patients with Sepsis and Acute Myeloid Leukemia: A National Inpatient Sample Analysis
Source: J Clin Med. 2025 Mar 25;14(7):2243. doi: 10.3390/jcm14072243 (PMC11989237; doi:10.3390/jcm14072243)
Supplement: Supplementary file 1 [file jcm-14-02243-s001.zip › jcm-3501332-supplementary.pdf]

Supplementary Materials:

Table S1: List of ICD-10 codes utilized in the present study

Diagnosis/Procedure	ICD 10 codes
Sepsis	A021, A227, A267, A327, A400, A401, A403, A408, A409, A4101, A4102, A411, A412, A413, A414, A4150, A4151, A4152, A4153, A4159, A4181, A4189, A419, A427, A5486, B377, R6520
Acute myeloid leukemia	C92.00, C92.01, C92.02
Acute kidney injury	N17
Arterial Hypertension	H35031, H35032, H35033, H35039, I10, I110, I119, I674, O111, O112, O113, O119, O114, O115, O1001, O1002, O1003, O10111, O10112, O10113, O10119, O161, O162, O163, O164, O165, O169, I160, I161, I169
Diabetes mellitus	E10.0, E10.1, E10.6, E10.8, E10.9, E11.0, E11.1, E11.6, E11.8, E11.9, E12.0, E12.1, E12.6, E12.8, E12.9, E13.0, E13.1, E13.6, E13.8, E13.9, E14.0, E14.1, E14.6, E14.8, E14.9
Diabetes mellitus with complications	E10.2–E10.5, E10.7, E11.2–E11.5, E11.7, E12.2–E12.5, E12.7, E13.2 E13.5, E13.7, E14.2–E14.5, E14.7
Hyperlipidemia	E78
Congestive heart failure	I09.9, I11.0, I13.0, I13.2, I25.5, I42.0, I42.5–I42.9, I43.x, I50.x, P29.0
Peripheral vascular disease	I70.x, I71.x, I73.1, I73.8, I73.9, I77.1, I79.0, I79.2, K55.1, K55.8, K55.9, Z95.8, Z95.9

Coronary artery disease	I25, I254
Cardiac Arrhythmia	I44.1–I44.3, I45.6, I45.9, I47–I49, R00.0, R00.1,<br>R00.8, T82.1, Z45.0, Z95.0
Cerebrovascular disease	I69, G454, G458, G459, I672, I679, I6782, I6789, I6781, Z8673
Chronic obstructive pulmonary disease	I27.8, I27.9, J40.x–J47.x, J60.x–J67.x, J68.4, J70.1, J70.3
Moderate/Severe liver disease	I85.0, I85.9, I86.4, I98.2, K70.4, K71.1, K72.1, K72.9, K76.5, K76.6, K76.7
Rheumatological disorders	M05.x, M06.x, M31.5, M32.x–M34.x, M35.1, M35.3, M36.0
Anemia	D51, D52, D53, D500, D508, D509
Obesity	E66, Z6830, Z6831, Z6832, Z6833, Z6834, Z6835, Z6836, Z6837, Z6838, Z6839, Z6841, Z6842, Z6843, Z6844, Z6845, Z684
Smoking	F17, Z720
Tumor lysis	E883

Fluid and electrolyte disorders	E87.0, E87.1, E87.2, E87.3, E87.4, E87.5, E87.6, E87.7, E87.8
Septic shock	R65.21
Vasopressor use	3E030XZ, 3E033XZ, 3E040XZ, 3E043XZ, 3E050XZ, 3E053XZ, 3E060XZ, 3E063XZ
Mechanical ventilation	5A1935Z, 5A1945Z, 5A1955Z
Mechanical ventilation >96 hours	5A1955Z, Z9911
